# Can the foregut nematode *Haemonchus contortus* and medicinal plants influence the fecal microbial community of the experimentally infected lambs?

**DOI:** 10.1371/journal.pone.0235072

**Published:** 2020-06-23

**Authors:** Dominika Mravčáková, Svetlana Kišidayová, Anna Kopčáková, Peter Pristaš, Jana Pisarčíková, Magda Bryszak, Adam Cieslak, Marián Várady, Zora Váradyová

**Affiliations:** 1 Institute of Animal Physiology, Centre of Biosciences, Slovak Academy of Sciences, Košice, Slovak Republic; 2 Department of Animal Nutrition, Poznan University of Life Sciences, Poznan, Poland; 3 Institute of Parasitology, Slovak Academy of Sciences, Košice, Slovak Republic; Beni Suef University, Faculty of Veterinary Medicine, EGYPT

## Abstract

The abomasal parasitic nematode *Haemonchus contortus* can influence the abomasal microbiome of the host. On the other hand, no information occurs on the influence of the parasite on the hindgut microbiome of the host. We evaluated the impact of *Haemonchus contortus* on the fecal microbial community of the experimentally infected lambs treated with a mixture of medicinal herbs to ameliorate the haemonchosis. Twenty-four female lambs were divided into four groups: infected animals (Inf), infected animals supplemented with a blend of medicinal herbs (Inf+Herb), uninfected control animals (Control), and uninfected animals supplemented with medicinal herbs (C+Herb). Inf and Inf+Herb lambs were infected orally with approximately 5000 L3 larvae of a strain of *H*. *contortus* susceptible to anthelmintics (MHco1). Herb blend (Herbmix) consisted of dry medicinal plants of *Althaea officinalis*, *Petasites hybridus*, *Inula helenium*, *Malva sylvestris*, *Chamomilla recutita*, *Plantago lanceolata*, *Rosmarinus officinalis*, *Solidago virgaurea*, *Fumaria officinalis*, *Hyssopus officinalis*, *Melisa officinalis*, *Foeniculum vulgare*, and *Artemisia absinthium*. Each animal was fed meadow hay and a commercial concentrate (600 + 350 g DM/d). Inf+Herb and C+Herb lambs were fed Herbmix (100 g DM/d and animal). Treatment lasted for 50 days. The fecal microbial fermentation parameters (short-chain fatty acids, ammonia, and pH) were evaluated at intervals of 0, 20, 32, and 50 days. The fecal eubacterial populations were evaluated by denaturing gradient gel electrophoresis (DGGE) at day 32 when *H*. *contortus* infection was the highest. No substantial effects of the *H*. *contortus* infection and the herbal treatment on fecal microbial fermentation parameters and fecal eubacterial populations were observed. Evaluation of DGGE patterns by Principal component analysis pointed to the tendency to branch the C+Herb group from the other experimental groups on Day 32. The results indicate that hindgut microbial activity was not disturbed by *H*. *contortus* infection and herbal treatment.

## Introduction

*Haemonchus contortus* is the most pathogenic gastrointestinal parasitic nematode of small ruminants, which adult worms attach to abomasal mucosa and feed on the blood. Young animals usually carry more onerous parasite burdens than adult animals resulting in reduced animal production and eventually deaths [1]. Chemoprophylaxis by repeated application of anthelmintics increases the risk of residues in food products and the development of anthelmintic resistance [2–4]. Medicinal herbs contain bioactive compounds with a broad activity toward human cells, bacteria, fungi, viruses, and parasites. Currently, the use of medicinal herbs with bioactive properties is an alternative strategy to control intestinal nematode populations [5,6]. Our recent studies showed that unique herbal blends might attenuate the severity of haemonchosis [7–9]. The complex interactions that occur between gastrointestinal parasitic helminths and commensal bacteria are essential for the cross-talk between host and parasite establishment, and mucosal immune system development [10].

Furthermore, *H*. *contortus* egg production is strongly correlated with abomasal pH values in sheep, suggesting that interactions among the host, gut microbiome, and the parasite are invoved [11–13]. Studies deal with the effects of helminth parasites on abomasal microbes of ruminants showed marked differences in the microbiome structure between infected and untreated animals [13,14]. The studies on non-ruminant animals showed that helminths could influence hindgut microbiome [15–17]. Although the *H*. *contortus* is foregut (abomasal) parasite, we cannot exclude that the presence and activities of the parasite can also influence the hindgut microbiome. However, no study deals with the effects of helminth parasites on the ruminant hindgut microbiome as a whole.

We assumed that the *H*. *contortus* infection might have an impact on the sheep fecal microbial fermentation parameters and sheep fecal eubacterial population. Our recent study showed the *in vitro* ovicidal and larvicidal effects of extracts of individual components of our unique herbal blend Herbmix [9]. In vivo, egg production by *H*. *contortus* females was decreased in the Inf + Herb group after day 32, which could be attributed to a possible deworming effect of the herbal treatment by Herbmix [7]. Herbmix influenced positively the lamb body-weights in the Inf + Herb group [7]. In the present study, we hypothesized that the dietary dry medicinal herb mixture Herbmix could also ameliorate likely hindgut microbial changes caused by *H*. *contortus*.

## Materials and methods

### Experimental design, lambs and treatments

The Ethics Committee of the Institute of Parasitology of the Slovak Academy of Sciences approved animal use and experimental design under the European Community guidelines (EU Directive 2010/63/EU for animal experiments). The lambs used in the present study were part of a larger *in vivo* experiment to investigate the effect of a mixture medicinal herbs (Herbmix) on ruminal fermentation, parasitological status and hematological parameters of the lambs experimentally infected with *Haemonchus contortus* and had been described in more detail previously [7]. Twenty-four female lambs (Improved Valachian, 3–4 months of age, initial body weights of 12.5 ± 1.64 kg) were housed in standard stalls for 15 d for acclimatization to feeding, with free access to water. Young animals of 3–4 months of age are more sensitive to *H*. *contortus* infection than older and adult animals [18]. Each animal was fed meadow hay (600 g DM/d) and a commercial concentrate (350 g DM/d; composed of 70% barley, 22% soybean meal, 4.8% wheat bran, 0.5% bicarbonate and 2.7% mineral–vitamin premix). The lambs were then randomly divided into four groups (n = 6/group, one stall per group): animals infected by *H*. *contortus* (Inf), infected animals supplemented with Herbmix (Inf+Herb), uninfected control animals (C) and uninfected animals supplemented with Herbmix (C+Herb). Inf and Inf+Herb lambs were infected orally with approximately 5000 L3 larvae of a strain of *H*. *contortus* susceptible to anthelmintics (MHco1) at day zero (D0). Inf+Herb and C+Herb lambs were fed Herbmix (100 g DM/d/animal) beginning from D0. Blend of dry medicinal plants labeled as Herbmix (non-commercial product) contained 8.55% each of *Althaea officinalis*, *Petasites hybridus*, *Inula helenium*, *Malva sylvestris*, *Chamomilla recutita*, *Plantago lanceolata*, *Rosmarinus officinalis*, *Solidago virgaurea*, *Fumaria officinalis*, *Hyssopus officinalis* and *Melisa officinalis*, 5% *Foeniculum vulgare* and 1% *Artemisia absinthium*. The individual components of Herbmix were obtained from commercial sources (AGROKARPATY, Plavnica, Slovak Republic and BYLINY Mikeš s.r.o., Číčenice, Czech Republic). Herbmix was stable throughout the experiment, daily mixed with commercial concentrate and supplemented to diets. The chemical compositions of the meadow hay, commercial concentrate, individual medicinal herbs, and Herbmix are given in Table 1. Samples of feces were collected from each animal at day 0, 20, 32, and 50 to measure pH, ammonia, and short-chain fatty acids (SCFA). Samples of feces for microbial 16S-PCR-DGGE analysis were collected from each animal on day 32. Feces samples were frozen at -60°C until molecular analysis. Day 32 was selected based on the evaluation of lambs parasitological status when the fecal egg output was the highest in the Infected group [7]. We assumed that the developed infection at day 32 might have an impact on the fecal microbial fermentation parameters and fecal eubacterial population. Animals were slaughtered at the end of the experiment in an abattoir at the Institute of Animal Physiology (Košice, № SK U 07016).

**Table 1:** Nutrient composition of diet substrates (n = 3).

Item	DM	aNDF	ADF	CP	N	Ash	IVDMD
	(g/kg)	(g/kg DM)					
Herbmix	896	479	345	151	24	98	570
Meadow hay	895	789	435	61	10	47	620
Concentrate	880	152	56	215	34	68	805

DM, dry matter; aNDF, neutral detergent fiber; ADF, acid detergent fiber; CP, crude protein; N, nitrogen; IVDMD, in vitro dry-matter digestibility.

### Chemical analysis of dietary substrates and feces microbial products

The dietary substrates (individual medicinal herbs, Herbmix, meadow hay, and commercial concentrate) were analyzed in triplicate by standard procedures [19], and they are shown in Table 1.

The DM content was obtained by drying the samples at 105°C for at least 24 h in an oven (method no. 930.15). The total ash content of the samples was determined by ashing overnight at 550°C (method no. 942.05) in a muffle furnace. Nitrogen (N) content (method no. 968.06) was determined using a FLASH 4000 (Thermo Fisher Scientific, Cambridge, UK). Crude-protein content was calculated by multiplying the total N content by 6.25 (method no. 990.03). The acidic-detergent fiber (ADF) and neutral-detergent fiber (aNDF) contents were analyzed as described by [20] using an ANKOM 2000 (ANKOM Technology, Macedon, USA) with heat-stable α-amylase. In vitro dry matter digestibility (IVDMD) of substrates was estimated according to [21]. Feces microbial activity patterns (pH, ammonia, and short-chain fatty acids) were measured in fresh feces, according to [21]. A liquid chromatograph Ultimate 3000 HPLC system (Dionex, Sunnyvale, CA, USA) was used for separation of six secondary plant metabolites: gallic acid (GA), rutin (RU), diosmin (DI), hesperidin (HE), quercetin (QU) and kaempferol (KA) according to [9]. The contents of flavonoids in Herbmix were 30.3 (RU, 4.05, DI, 13.01, HE, 8.06, QU, 4.69, and KA, 0.51) and phenolic acids 0.44 g/kg DM (as GA).

### DNA isolation, PCR amplification, and DGGE analysis

The total DNA from frozen feces samples was extracted by QIAamp DNA Stool Mini Kit (Qiagen, Valencia, CA, USA. Isolated DNA was used as a template for PCR amplification of 16S rRNA gene fragments.

All PCR reactions were performed in a 50 μl PCR mixture containing 1 μl the substrate of DNA, 1 x PCR buffer, 2 mmol/l MgCl2, 1 μl of a 200 μmol/l of each dNTP, 1.25 U Platinum Taq DNA polymerase (Invitrogen, CA USA) and 25 pmol each primer using MJ Mini thermal cycler (Bio-Rad Laboratories, USA).

In the first round of PCR universal primers fD1 (5´-AGA GTT TGA TCC TGG CTC AG-3´) and rP2 (5´-ACG GCT ACC TTG TTA CGA CTT-3´) [22] were used to amplify a 1500 bp region of 16S rDNA gene. PCR conditions were as follows: 94°C for 5 min followed by 35 cycles of 94°C for 1 min, 52°C for 1 min, 72°C for 1 min 30 sec, 72°C for 5 min. The obtained 16S rDNA fragments were subsequently used as a template for the second round of PCR using specific bacterial primers GC-clamp-968f (5´- CGC CCG GGG CGC GCC CCG GGC GGG GCG GGG GCA CGG GGG GAA CGC GAA GAA CCT TAC—3´) and 1401r (5´- CGG TGT GTA CAA GAC CC– 3´) [23].

The cycling conditions were 94°C for 5 min, 9 cycles of 94°C for 1 min, 45°C for 1 min, 72°C for 1 min; 14 cycles of 94°C for 1 min, 60°C for 1 min, 72°C for 1 min followed by final extension at 72°C for 10 min. PCR products were detected by 1% agarose gel electrophoresis containing ethidium bromide and photographed using the Gel Logic 212 PRO imaging system (Carestream, NY USA).

PCR products generated with GC–clamp-968f and 1401r primers were subjected to DGGE analysis. DGGE was performed using DCodeTM Universal Mutation Detection System (Bio-Rad Laboratories, Hercules, CA USA). The round II PCR reaction products in a total volume of 45 μl were loaded onto 8% (w/v) polyacrylamide gel (40% Acrylamide-Bis 37.5:1) in 1 x TAE (40 mM Tris, 20 mM acetate, 1mM EDTA) containing a linear denaturing gradient ranging from 30–60% denaturant (100% denaturant solution consists of 7 M urea and 40% formamide). Electrophoresis was run for 17h at a constant voltage of 50V and a temperature of 60°C. After electrophoresis, the gel was incubated for 20 min in ethidium bromide (0.5 g/ml), rinsed for 20 min in distilled water, and photographed with UV transillumination using the Gel Logic Imaging System (Carestream, NY USA).

### Statistical analysis

DGGE fingerprints were transformed, and dendrogram and population variability indices (Species richness, Shannon-Wiener diversity, and Evenness) were counted according to [24]. The one-way analysis with Bonferroni Multiple Comparison Test was used to evaluate of population variability indices of DGGE profiles. The differences in fecal microbial parameters (pH, ammonia, and SCFA) were evaluated by two-way analysis of variance and Bonferroni Multiple Comparison Test as a repeated measures mixed model of four animal groups (Inf, Inf+Herbmix, Control, Control+Herbmix) and sampling days (GraphPad Software, Inc. San Diego, CA, USA) according to [7]. Treatment effects were determined to be significant at *P < 0*.*05*.

## Results

The effects of Time on pH of feces, and effects of Time, and Treatment × Time on SCFAs, acetate, propionate, and butyrate were observed in feces (Table 2).

**Table 2:** Microbial activity indices in the feces of lambs infected with *H*. *contortus* (Inf), infected lambs supplemented with Herbmix (Inf + Herb), uninfected lambs (C), and uninfected lambs supplemented with Herbmix (C + Herb) (n = 6).

Parameter	Day	Inf	Inf+Herb	C	C+Herb	SD	Significance of effects:				
							Treatment			Time	Treatment × time
							Inf vs. Inf + Herb	Inf vs. C	Inf vs. C + Herb		
pH	0	7.23	7.24	7.41	7.37	0.363	Ns	Ns	Ns	*	Ns
	20	7.23	7.12	7.07	7.18	0.431	Ns	Ns	Ns		
	32	7.13	7.34	7.04	7.36	0.265	Ns	Ns	Ns		
	50	7.67	7.47	7.54	7.14	0.192	Ns	Ns	Ns		
Ammonia	0	216	220	234	212	30.5	Ns	Ns	Ns	Ns	Ns
(mg/l)	20	200	203	216	225	50.4	Ns	Ns	Ns		
	32	211	214	227	220	38.7	Ns	Ns	Ns		
	50	220	214	235	249	29.6	Ns	Ns	Ns		
SCFAs	0	66.2	72.8	56.9	50.0	13.34	Ns	Ns	Ns	**	*
(mmol/l)	20	65.5	64.3	63.6	62.0	15.01	Ns	Ns	Ns		
	32	52.2	44.3	59.3	53.4	9.07	Ns	Ns	Ns		
	50	58.7	54.2	44.7	56.4	8.92	Ns	Ns	Ns		
Acetate	0	82.0	84.6	85.1	86.2	3.42	Ns	Ns	*	**	*
(mol%)	20	82.1	86.9	80.2	82.6	3.04	Ns	Ns	Ns		
	32	80.6	84.2	79.2	82.6	2.63	Ns	Ns	Ns		
	50	82.8	79.5	80.7	84.0	2.75	Ns	Ns	Ns		
Propionate	0	13.8	11.6	11.7	10.0	2.04	Ns	Ns	*	Ns	Ns
(mol%)	20	12.6	9.35	13.7	10.6	2.91	Ns	Ns	Ns		
	32	12.7	9.75	12.8	11.5	1.93	Ns	Ns	Ns		
	50	14.3	9.74	12.9	10.9	2.25	Ns	Ns	Ns		
Butyrate	0	2.57	2.88	2.62	2.30	0.513	Ns	Ns	*	**	***
(mol%)	20	2.29	4.51	4.73	4.64	2.034	Ns	Ns	Ns		
	32	5.24	3.71	3.77	4.43	1.867	Ns	Ns	Ns		
	50	5.88	3.67	5.90	4.20	1.364	Ns	Ns	Ns		
Isobutyrate	0	0.58	0.27	0.50	0.61	0.138	Ns	Ns	Ns	Ns	Ns
(mol%)	20	0.44	0.11	0.35	0.46	0.065	Ns	Ns	Ns		
	32	0.62	0.16	0.20	0.64	0.038	Ns	Ns	Ns		
	50	0.84	0.21	0.37	0.67	0.098	Ns	Ns	Ns		
Valerate	0	0.71	0.26	0.42	0.25	0.086	Ns	Ns	Ns	Ns	Ns
(mol%)	20	0.69	0.40	0.53	0.83	0.163	Ns	Ns	Ns		
	32	0.47	0.48	0.57	0.52	0.132	Ns	Ns	Ns		
	50	0.87	0.56	0.73	0.82	0.075	Ns	Ns	Ns		
Isovalerate	0	0.62	0.56	0.46	0.86	0.054	Ns	Ns	Ns	Ns	Ns
(mol%)	20	0.44	0.47	0.77	0.60	0.157	Ns	Ns	Ns		
	32	0.72	0.81	0.64	0.53	0.132	Ns	Ns	Ns		
	50	0.87	0.76	0.85	0.82	0.075	Ns	Ns	Ns		
Caproate	0	0.09	0.04	0.13	0.10	0.088	Ns	Ns	Ns	Ns	Ns
(mol%)	20	0.06	0.07	0.12	0.11	0.083	Ns	Ns	Ns		
	32	0.08	0.14	0.05	0.09	0.062	Ns	Ns	Ns		
	50	0.05	0.04	0.13	0.10	0.044	Ns	Ns	Ns		

Ns, not significant

*P < 0.05

**P < 0.01

***P < 0.001; Values are means ± standard deviation.

The differences were observed at the beginning of the experiment (day 0) between the infected group and C+Herb group in the case of acetate, propionate, and butyrate. At Day 32 of the experiment, no differences were observed. Evaluation of population variability of DGGE patterns revealed no changes in eubacterial fecal population richness, diversity (Shannon index), and evenness (Pielou index) at the Day 32 (Table 3).

**Table 3:** Evaluation of population variability of DGGE profiles of lamb feces eubacteria at day 32 after *H*. *contortus* infection and treated with a blend of medicinal plants (Herbmix) (n = 6).

Item	Control	Control + Herbmix	Infected	Infected + Herbmix	Statistics
Species richness	10 ± 1.2	10 ± 1.5	8 ± 1.3	11 ± 0.9	Ns
Diversity (Shannon index)	1.93 ± 0.148	1.96 ± 0.199	1.65 ± 0.237	2.09 ± 0.106	Ns
Evenness (Pielou index)	0.82 ± 0.026	0.89 ± 0.040	0.83 ± 0.051	0.88 ± 0.021	Ns

The number of bands was evaluated in DGGE profiles; Values are means ± standard error; Ns, not significant; Herbmix, a blend of medicinal plants.

**Fig 1:**
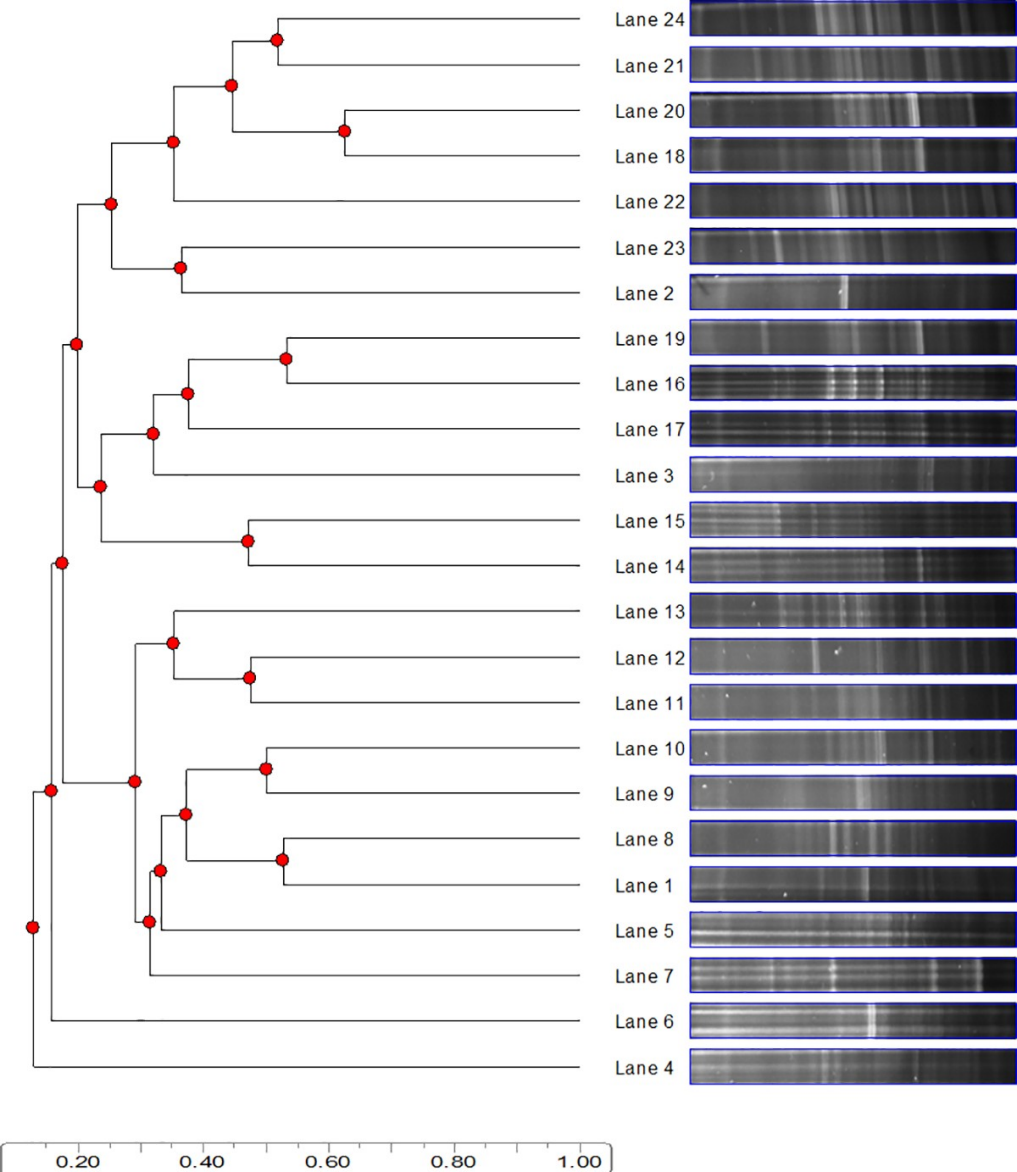
Principal component analysis of denaturing gradient gel electrophoresis (DGGE) patterns of eubacterial community in feces of lambs infected with *Haemonchus contortus* and treated with a blend of medicinal plants (Herbmix). Infected group (Lanes 1-6); Infected + Herbmix (Lanes 7-12); Control group (Lanes 13-18); Control + Herbmix (Lanes 19-24).

However, evaluation of DGGE patterns by Principal component analysis pointed to the tendency to branch the C+Herb group (Lines 18, 20–24) from the other experimental groups on Day 32 (Fig 1).

## Discussion

In the present study, the results are a part of the comprehensive experiment investigated the effects of a dry mixture of medicinal herbs (Herbmix) on parasitological status, hematological parameters, inflammatory response, antioxidant status, and mineral status in lambs experimentally infected with *H*. *contortus* [7]. The recent studies point to the interactions within helminths, microbes, and their hosts [25,26]. Intestinal helminths can modulate antioxidant status and host immune response to establish and persist from months to years in their host [27–29]. On the other hand, the prokaryotes can modulate a parasite’s colonization success, propagation, and infectivity, shifting it within the parasitism-mutualism scale [30,31]. It is hypothesized that the long coevolution of vertebrate hosts with their microbes and parasitic helminths is fundamental for the host immune system regulation [32]. Some researchers propose the association of immune disorders with the deficit of helminth-induced immunomodulation [27,33–35].

Most of the studies dealing with the relationship of helminth gut parasites and gut prokaryotes use hindgut digestion animals, preferably rodent animal models and humans. The studies dealing with the relationship of helminth gut parasites and gut prokaryotes of ruminants are scarce [13,14,36,37]. A study on sheep showed that ruminal and abomasal microbiome was altered during acute as well as 50 days of the *H*. *contortus* infection, which probably facilitates parasite survival and reproduction [13]. The similar observations were described on the caprine abomasal microbiome after 50 days of the *H*. *contortus* infection [14]. On the other hand, some treatments as immunization against helminth abomasal parasite, *Ostertagia ostertagi*, had minimal impact on calf abomasal microbial ecosystem [36].

Our hypothesis assuming influence of medicinal herb mixtures supplemented to the diet of lambs infected by *H*. *contortus* on *fecal* eubacterial population and microbial fermentation has not been however confirmed. Our present study has shown no effects of *Haemonchus* infection and herbal treatments on fecal eubacteria of sheep, as was revealed by PCR-DGGE fingerprinting. The similar results were observed in the study of Mravčáková et al. [8]. However, DGGE fingerprinting can evaluate just the most dominant members of the prokaryotes, which means that they gain the level of at least 3% of the total population [24,38,39]. Despite of that general DGGE limitation, our limited sample size and the sequence of the excised DGGE bands, the DGGE fingerprinting is sufficiently robust and valuable as the first line assay to a quick evaluation of microbial diversity changes [38,40]. However, to evaluate the diversity changes at a larger scale, the sequencing methods are essential. The influence of diet on fermentation patterns and the microbial population is another factor that could modulate gut parasitic infections. There is increasing discussion on the therapeutic potential of diets [41]. Studies on rumen fermentation and gut microbiome reveal the ambiguous effects of medicinal herb supplements [8,39,42–45]. It seems that response and adaptation to herbal bioactive compounds, fermentation kinetics, and diet composition are among the significant factors contributing to the inconsistent efficacy of medicinal herb supplements [46]. However, interplay with host, herbal supplements, and parasitic infection was shown in vitro and in vivo [7–9,47]. It seems that medicinal plant supplements could influence nematode infection through modulation of host antioxidant status and some immunological parameters [7,8]. However, the minimal effects of the supplement of the blend of medicinal herbs on fecal fermentation patterns and the fecal eubacterial population were observed in our study. It seems that adding the herbal supplement to the diets of infected lambs did not affect the composition of the fecal as well as ruminal eubacterial communities in neither this nor other in vivo studies in a substrate-specific manner [8,48].

## Conclusions

We can conclude that primary site of *Haemonchus* infection effects is abomasum with minimum impact on hindgut dominant microbial members and microbial fermentation patterns. The experimental herbal blend had no significant impact on fecal eubacteria and fecal fermentation activities. However, the sequencing methods could reveal changes in hindgut minor prokaryote populations because of nematode infection.
